# Vitreous amyloidosis with autonomic neuropathy of the digestive tract associated with a novel transthyretin p.Gly87Arg variant in a Bangladeshi patient: a case report

**DOI:** 10.1186/s13256-017-1407-z

**Published:** 2017-08-13

**Authors:** Benjamin Terrier, Magali Colombat, Caroline Beugnet, Astrid Quéant, Jonathan London, Jean-Baptiste Daudin, Claire Le Jeunne, Luc Mouthon, Dominique Monnet, Cécile Cauquil, Catherine Lacroix, David Adams, Antoine Brézin, Sophie Valleix

**Affiliations:** 1 Department of Internal Medicine, Referral Center for Systemic and Autoimmune Diseases, Hôpital Cochin, AP–HP, Université Paris-Descartes, 75014 Paris, France; 2 Department of Pathology, Institut Universitaire du Cancer, Toulouse, France; 3 Université Paris-Descartes, Sorbonne Paris Cité, Faculté de Médecine Paris, AP-HP, Laboratoire de Génétique Moléculaire, Hôpital Necker-Enfants Malades, Paris, France; 4 Department of Ophthalmology, Hôpital Cochin, AP–HP, Université Paris-Descartes, 75014 Paris, France; 5 Department of Neurology, Referral Center for familial amyloid polyneuropathy (NNERF), Hôpital Bicêtre, AP–HP, Université Paris-Sud, 94270 Le Kremlin-Bicêtre, France; 6 INSERM, UMR_1163, IHU Imagine–Institut des Maladies Génétiques, Laboratoire de Génétique Ophtalmologique, Paris, France; 7 Génétique Moléculaire, Hôpital Necker-Enfants Malades, Paris, France

**Keywords:** Amyloidosis, Bangladeshi, Transthyretin, Vitreous amyloidosis

## Abstract

**Background:**

Hereditary transthyretin amyloidosis is an autosomal dominant inherited disorder, first described in families with sensorimotor and autonomic neuropathy. Since its first description, more than 120 amyloidogenic transthyretin mutations have been reported with various geographic distributions and associated with a wide range of phenotypes involving the peripheral nerve, the heart, the gastrointestinal tract, the eyes, the central nervous system, or the kidneys. In some cases of transthyretin amyloidosis, the first clinical manifestation is vitreous opacity.

**Case presentation:**

A 46-year-old Bangladeshi woman presented with vitreous amyloidosis and progressive autonomic neuropathy of the digestive tract as initial clinical manifestations, with no clinical evidence of cardiac, renal, central nervous system, or peripheral nerve dysfunction. A novel transthyretin mutation, p.Gly87Arg, was identified in the heterozygous state in this proband of Bangladeshi origin. Histological examination of accessory salivary glands and gastric biopsies revealed Congo-red-positive deposits. Laser microdissection of salivary gland Congo-red deposits and tandem mass spectrometry-based proteomic analysis identified the mutated transthyretin peptide containing the arginine residue at position 87 of the mature protein.

**Conclusions:**

Vitreous amyloidosis should be considered a differential diagnosis of uveitis, in particular transthyretin amyloidosis. Proteomics data from our case, consistent with the genetic findings, highly suggests that this new p.Gly87Arg variant is amyloidogenic. Here, we described the second case of transthyretin amyloidosis reported in a Bangladeshi patient.

## Background

Hereditary transthyretin amyloidosis (ATTR amyloidosis), Online Mendelian Inheritance in Man (OMIM) 105210, is an autosomal dominant inherited disorder, first described in families from Northern Portugal with sensorimotor and autonomic neuropathy, and associated with the Val30Met (p.Val50Met) transthyretin (TTR) mutation [1]. Since its first description in the Portuguese population, more than 120 amyloidogenic TTR mutations have been reported with various geographic distributions and associated with a wide range of phenotypes involving the peripheral nerve, the heart, the gastrointestinal tract, the eyes, the central nervous system, or the kidneys [2]. In some cases of TTR amyloidosis, the first clinical manifestation is vitreous opacity, often associated with leptomeningeal amyloidosis. We describe a case of a Bangladeshi patient presenting with ATTR amyloidosis with vitreous amyloid deposition and autonomic neuropathy of the digestive tract as initial clinical manifestations, and associated with a novel amyloidogenic TTR mutation, Gly67Arg (p.Gly87Arg).

## Case presentation

**Fig. 1:**
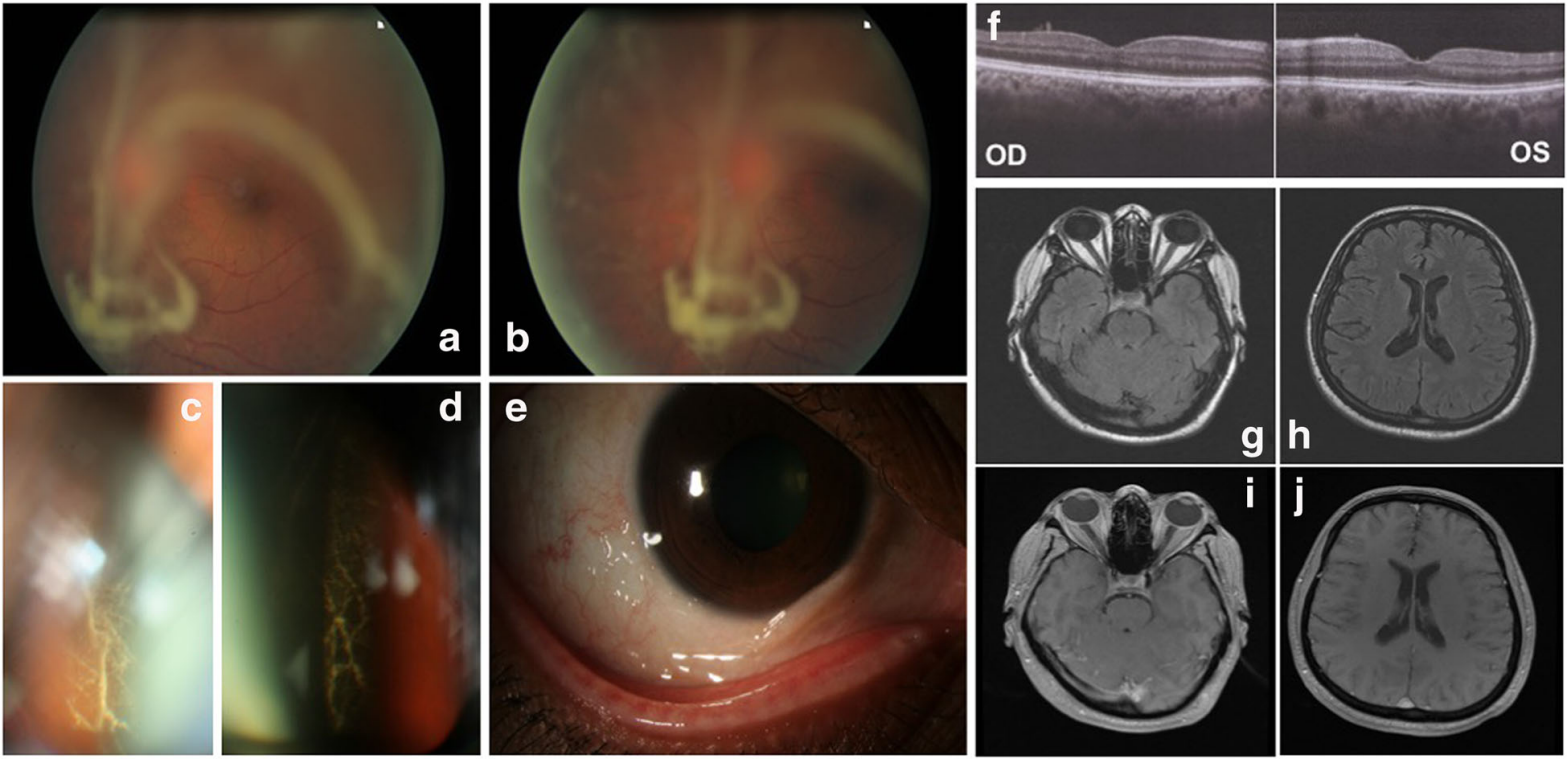
Vitreous amyloidosis. An ocular examination revealed accumulation of extensive fibrillogranular deposits in the vitreous consistent with amyloid deposits (panels **a**, **b**, **c** and **d**), and conjunctivochalasis (panel **e**). Optical coherence tomography showed normal retinal appearance with a normal macular and foveal thickness (panel **f**). Magnetic resonance imaging of the brain revealed no leptomeningeal involvement associated with vitreous amyloidosis on T2-weighted fluid-attenuated inversion recovery sequences (panels **g** and **h**) and gadolinium-enhanced T1-weighted sequences (panels **i** and **j**). *OD* right eye, *OS* left eye

**Fig. 2:**
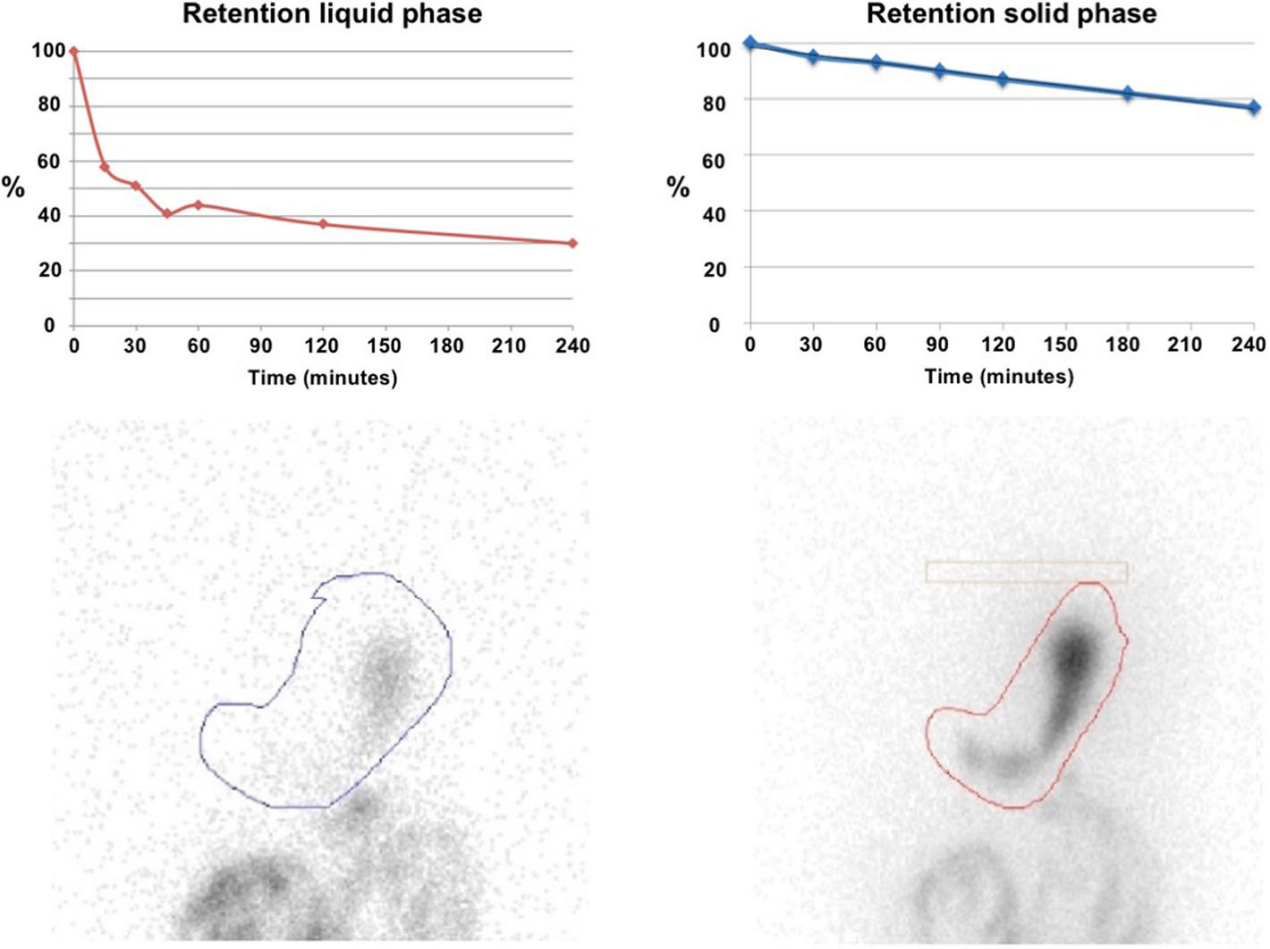
Gastric scintigraphy. The test consisted of an exploration of both the solid (ovalbumin with ^99m^Tc-labeled colloids) and the liquid (^111^In diethylene triamine pentaacetic acid diluted in water) phases of gastric emptying (*left* panel for liquids and *right* panel for solids). The parameters used for the result interpretation were the half-time emptying (T1/2) and the residual activity at 4 hours (activity/time curves for liquid and solid). In this case, there was a very slow and gradual reduction of the tracer in the stomach. The residual activity remains very high 4 hours after ingestion: 30% at 4 hours (N< 10%) for liquid, and 77% (N< 10%) for solids

**Fig. 3:**
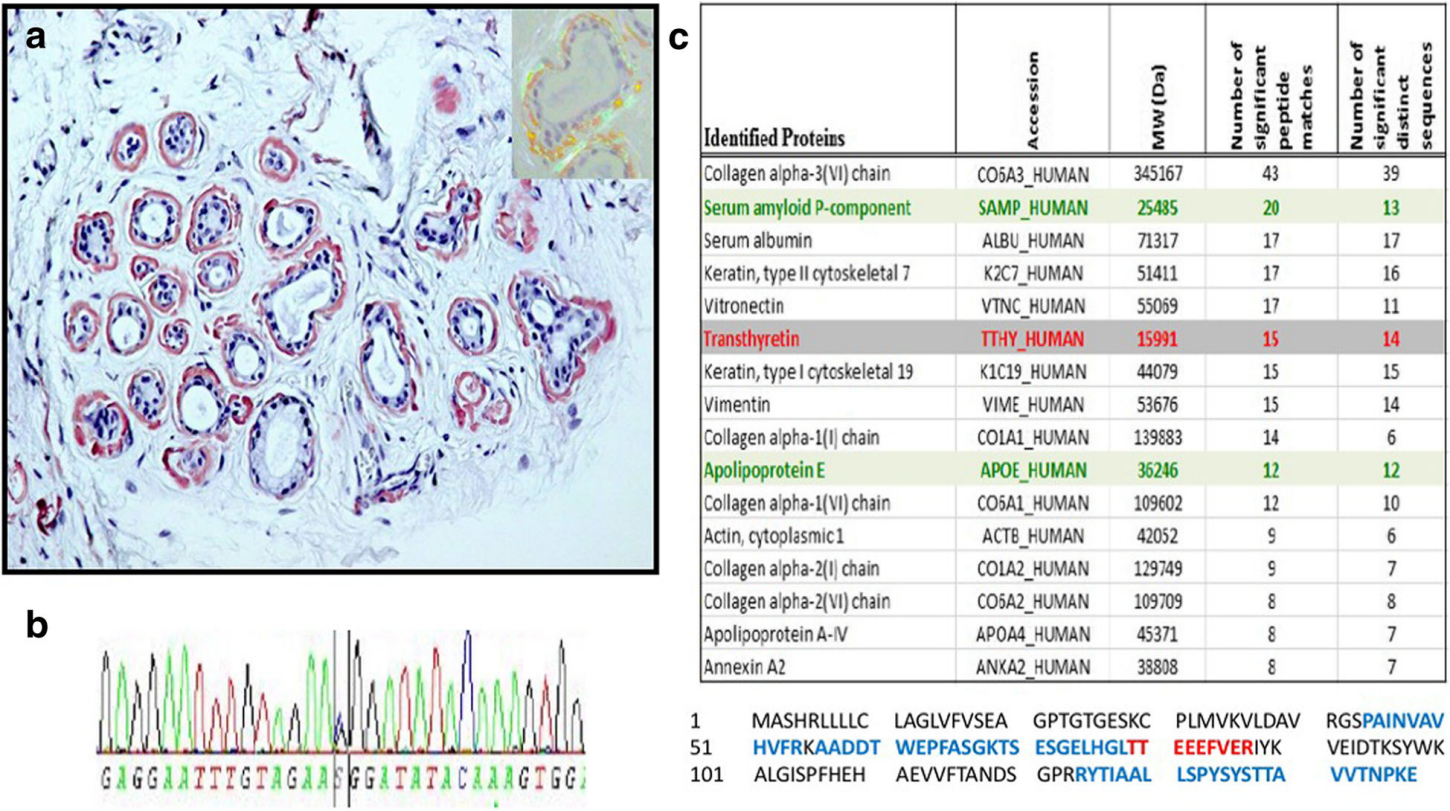
Analysis of amyloidosis deposits. The Congo-red stain on minor salivary gland biopsy (panel **a**) showed deposits localized around the acini and in the interstitium with an apple-green birefringence under polarized light (*insert*). Partial sequencing electropherogram showing the c.259 G>C in exon 3 of transthyretin, resulting in replacement of glycine with arginine at position 87 of the mature protein (Panel **b**), that is, p.Gly87Arg (Gly67Arg). Proteomic profile of the amyloid deposits using tandem mass spectrometry analysis identified transthyretin as the dominant amyloid protein (panel **c**). Transthyretin was associated with several proteins known to be associated with amyloid deposits such as serum amyloid P-component and apolipoprotein E. The protein sequence coverage shown in bold *blue* was 45%, and the mutated peptide is shown in bold *red*

A 46-year-old Bangladeshi woman presented with a 36-month history of blurred vision with floaters. This proband was otherwise asymptomatic without weight loss and with no relevant past medical history. She had no familial history of amyloidosis. Visual acuity was 7/10 P2 for her right eye and 9/10 P2 for her left eye. Intraocular pressure was normal in both eyes (15 mmHg). No pars plana vitrectomy was performed. A slit-lamp biomicroscopy showed abnormal conjunctival vessels with keratoconjunctivitis sicca. No change of the pupillary shape and no amyloid deposition on the lens surface or at the pupillary border were noted. Light pupillary reflex was normal. Fundoscopy revealed yellowish-white “glass-wool” vitreous opacity in both eyes consistent with amyloid deposits (Fig. 1a–e). The macula and the optic disc appeared normal, and no retinal hemorrhages were detected. Optical coherence tomography (OCT) revealed a normal retinal morphology including in the area of the macula (Fig. 1f). An extensive evaluation, including extensive cardiac investigation, did not reveal other clinical features. Magnetic resonance imaging of her brain was normal, without any leptomeningeal involvement (Fig. 1g–j). She did not have carpal tunnel syndrome, and a neurological examination of her peripheral nerve system did not detect abnormalities in her upper and lower limbs. The results of nerve conduction studies were normal, but a distal leg skin biopsy detected loss of intra-epidermal nerve fiber density with a value of 0.29 fiber/millimeter, supporting the diagnosis of small fiber neuropathy [3]. She progressively experienced postural dizziness and vomiting, and gastric emptying scintigraphy confirmed gastroparesis with abnormal gastric retention: for liquids, 30% at 4 hours (N< 10%); and for solids, 77% (N<10%), demonstrating severe autonomic neuropathy (Fig. 2). A histological examination of accessory salivary glands (Fig. 3a) revealed Congo-red-positive deposits with an apple-green birefringence under polarized light in the interstitium and basement membranes of the acini. A biopsy of her stomach revealed perivascular amyloid deposits in the submucosa and the muscular circular layer. Direct sequencing of *TTR* gene revealed that she was heterozygous for a single nucleotide substitution c.259 G>C in exon 3, resulting in replacement of glycine with arginine at position 87 of the mature protein, that is, p.Gly87Arg (Gly67Arg; Fig. 3b). Conservation analysis of ten vertebrate species indicated that Glycine 87 is highly conserved in the TTR protein. Proteomic analysis performed on the formalin-fixed paraffin-embedded salivary gland tissue using laser microdissection followed by tandem mass spectrometry identified TTR as the dominant amyloid protein and identified the mutated TTR peptide (p.Gly87Arg) within the deposits, whereas the corresponding wild-type TTR peptide was not found (Fig. 3c). TTR was associated with several proteins known to be associated with amyloid deposits such as serum amyloid P-component and apolipoprotein E. In Fig. 3c, the protein sequence coverage shown in bold blue was 45%, and the mutated peptide is shown in bold red.

## Discussion

In the present report, we describe a novel amyloidogenic TTR mutation, p.Gly87Arg, associated with vitreous amyloidosis and autonomic neuropathy of the digestive tract in a Bangladeshi patient. ATTR amyloidosis was diagnosed based on the clinical findings, heterozygosity for the new TTR mutation, amyloid deposition in salivary glands and gastric tissue, and confirmation of the presence of the p.Gly87Arg variant within the deposits by tandem mass spectrometry-based proteomic analysis. Vitreous amyloidosis has been reported to be a differential diagnosis of uveitis, in particular TTR amyloidosis [4, 5]. It is the second description of ATTR in a Bangladeshi patient, with the first case being reported in a family with polyneuropathy associated with the Ile73Val TTR variant, without vitreous amyloidosis [6]. The p.Gly87Arg mutation is a new amyloidogenic TTR variant associated with vitreous deposition. Circulating TTR is mainly derived from the liver, but TTR is also synthesized by the retinal pigment epithelium of the eye [7] and choroid plexus of the brain [8], which can lead to vitreal and leptomeningeal amyloid deposition. Vitreous amyloid deposition, presumed to result from local synthesis of TTR in the eye, was reported with various TTR mutations and variably associated with leptomeningeal amyloid deposition [9]. It has been shown that in the case of Val30Gly, Tyr114Cys, Val30Met, and Ile84Ser TTR variants associated with vitreous amyloidosis, the TTR amyloid deposition consisted almost entirely of the variant protein with only a mild percentage of the wild-type TTR [10]. Here, we found that the amyloid fibrils were mainly composed of the p.Gly87Arg variant, albeit proteomic analysis was performed on salivary tissue and not directly from vitreous deposits.

## Conclusions

In the eye, vitreous amyloidosis has been reported as a differential diagnosis of uveitis, in particular TTR amyloidosis. We report a case of vitreous opacity and severe autonomic neuropathy of the digestive tract associated with a novel TTR amyloidogenic variant, p.Gly87Arg. This is the second reported case of ATTR amyloidosis in a Bangladeshi patient, further supporting that ATTR is a worldwide genetic disorder.

## References

[1] Planté-Bordeneuve V, Said G. Familial amyloid polyneuropathy. Lancet Neurol. 2011;10:1086–97. 10.1016/S1474-4422(11)70246-0 22094129

[2] Sekijima Y. Transthyretin (ATTR) amyloidosis: clinical spectrum, molecular pathogenesis and disease-modifying treatments. J Neurol Neurosurg Psychiatry. 2015;86:1036–43. 10.1136/jnnp-2014-308724 25604431

[3] Lauria G, Bakkers M, Schmitz C, Lombardi R, Penza P, Devigili G, et al. Intraepidermal nerve fiber density at the distal leg: a worldwide normative reference study. J Peripher Nerv Syst JPNS. 2010;15:202–7. 10.1111/j.1529-8027.2010.00271.x 21040142

[4] Coupland SE. The pathologist’s perspective on vitreous opacities. Eye Lond Engl. 2008;22:1318–29. 10.1038/eye.2008.31 18344965

[5] Seca M, Ferreira N, Coelho T. Vitreous Amyloidosis as the Presenting Symptom of Familial Amyloid Polyneuropathy TTR Val30Met in a Portuguese Patient. Case Rep Ophthalmol. 2014;5:92–7. 10.1159/000360790 PMC3985800 24748873

[6] Booth DR, Gillmore JD, Persey MR, Booth SE, Cafferty KD, Tennent GA, et al. Transthyretin Ile73Val is associated with familial amyloidotic polyneuropathy in a Bangladeshi family. Mutations in brief no. 158. Online. Hum Mutat. 1998;12:135. 10.1002/(SICI)1098-1004(1998)12:2<135::AID-HUMU10>3.0.CO;2-6 10694917

[7] Getz RK, Kennedy BG, Mangini NJ. Transthyretin localization in cultured and native human retinal pigment epithelium. Exp Eye Res. 1999;68:629–36. 10.1006/exer.1998.0646 10328977

[8] Schreiber G. The evolution of transthyretin synthesis in the choroid plexus. Clin Chem Lab Med. 2002;40:1200–10. 10.1515/CCLM.2002.210 12553420

[9] Liu T, Zhang B, Jin X, Wang W, Lee J, Li J, et al. Ophthalmic manifestations in a Chinese family with familial amyloid polyneuropathy due to a TTR Gly83Arg mutation. Eye Lond Engl. 2014;28:26–33. 10.1038/eye.2013.217 PMC3890754 24113303

[10] Liepnieks JJ, Wilson DL, Benson MD. Biochemical characterization of vitreous and cardiac amyloid in Ile84Ser transthyretin amyloidosis. Amyloid Int J Exp Clin Investig Off J Int Soc Amyloidosis. 2006;13:170–7. 10.1080/13506120600877003 17062384

